# Individual Assessment of Perioperative Brain Growth Trajectories in Infants With Congenital Heart Disease: Correlation With Clinical and Surgical Risk Factors

**DOI:** 10.1161/JAHA.122.028565

**Published:** 2023-07-08

**Authors:** Daniel Cromb, Alexandra F. Bonthrone, Alessandra Maggioni, Paul Cawley, Ralica Dimitrova, Christopher J. Kelly, Lucilio Cordero‐Grande, Olivia Carney, Alexia Egloff, Emer Hughes, Joseph V. Hajnal, John Simpson, Kuberan Pushparajah, Mary A. Rutherford, A. David Edwards, Jonathan O'Muircheartaigh, Serena J. Counsell

**Affiliations:** ^1^ Centre for the Developing Brain, School of Biomedical Engineering and Imaging Sciences King’s College London London United Kingdom; ^2^ Department for Forensic and Neurodevelopmental Sciences Institute of Psychiatry, Psychology and Neuroscience, King’s College London London United Kingdom; ^3^ Biomedical Image Technologies, Escuela Técnica Superior de Ingenieros (ETSI) de Telecomunicación Universidad Politécnica de Madrid and Centro de Investigación Biomédica en Red Bioengineering, Biomaterials and Nanomedicine (CIBER‐BBN) Madrid Spain; ^4^ Paediatric Cardiology Department Evelina London Children’s Healthcare London United Kingdom; ^5^ Medical Research Council Centre for Neurodevelopmental Disorders King’s College London London United Kingdom

**Keywords:** brain volumes, congenital heart disease, surgery, Magnetic Resonance Imaging (MRI)

## Abstract

**Background:**

Infants with congenital heart disease (CHD) are at risk of neurodevelopmental impairments, which may be associated with impaired brain growth. We characterized how perioperative brain growth in infants with CHD deviates from typical trajectories and assessed the relationship between individualized perioperative brain growth and clinical risk factors.

**Methods and Results:**

A total of 36 infants with CHD underwent preoperative and postoperative brain magnetic resonance imaging. Regional brain volumes were extracted. Normative volumetric development curves were generated using data from 219 healthy infants. *Z‐*scores, representing the degree of positive or negative deviation from the normative mean for age and sex, were calculated for regional brain volumes from each infant with CHD before and after surgery. The degree of *Z*‐score change was correlated with clinical risk factors. Perioperative growth was impaired across the brain, and it was associated with longer postoperative intensive care stay (false discovery rate *P*<0.05). Higher preoperative creatinine levels were associated with impaired brainstem, caudate nuclei, and right thalamus growth (all false discovery rate *P*=0.033). Older postnatal age at surgery was associated with impaired brainstem and right lentiform growth (both false discovery rate *P*=0.042). Longer cardiopulmonary bypass duration was associated with impaired brainstem and right caudate growth (false discovery rate *P*<0.027).

**Conclusions:**

Infants with CHD can have impaired brain growth in the immediate postoperative period, the degree of which associates with postoperative intensive care duration. Brainstem growth appears particularly vulnerable to perioperative clinical course, whereas impaired deep gray matter growth was associated with multiple clinical risk factors, possibly reflecting vulnerability of these regions to short‐ and long‐term hypoxic injury.

**Nonstandard Abbreviations and Acronyms**
dHCP: developing Human Connectome Project
FDR: false discovery rate

**Clinical Perspective**

**What Is New?**

- This is the first study to investigate individualized perioperative brain growth in infants with congenital heart disease by quantifying the degree to which an infant with congenital heart disease deviates from the typical population and how this deviation changes over time.
- Growth in cortical gray matter, white matter, cerebellum, brainstem, right lentiform, right thalamus, and total brain tissue volume is impaired in the immediate postoperative period.
- Infants requiring prolonged postoperative intensive care are at highest risk of impaired perioperative brain growth.

**What Are the Clinical Implications?**

- Later age at surgery, longer duration of cardiopulmonary bypass, and longer time on intensive care postoperatively are associated with impaired perioperative brainstem growth.
- Higher preoperative creatinine may be a modifiable preoperative clinical factor in infants with congenital heart disease as it is associated with reduced perioperative growth in the brainstem, caudate nuclei, and right thalamus.
- Extracerebral cerebrospinal fluid volume *Z*‐scores were significantly reduced after surgery, with absolute postoperative cerebrospinal fluid volumes being close to the typical mean, suggesting congenital cardiac surgery or catheterization may normalize extracerebral cerebrospinal fluid volumes in the immediate postoperative period.

Congenital heart disease (CHD) is the most common congenital malformation.^1^ Improvements in surgical techniques and perioperative care have resulted in most infants born with severe CHD surviving into adulthood.^2^ However, infants with CHD, particularly those who undergo early corrective or palliative surgical procedures, remain at an increased risk of neurodevelopmental impairments.^3^, ^4^ These neurodevelopmental impairments may arise from impaired early brain development or acquired brain injury. However, the mechanisms leading to impaired brain development in the perioperative period remain unclear and are likely to be multifactorial.

Many magnetic resonance imaging (MRI) studies have reported altered brain maturation in neonates with CHD before surgery. The pattern of altered brain development includes reduced global and regional volumes,^5^, ^6^, ^7^ impaired cortical gyrification,^8^, ^9^ altered white matter,^10^, ^11^ and cortical microstructure.^12^

Cardiac interventions, particularly surgery requiring cardiopulmonary bypass, have been associated with new brain injury on MRI,^13^, ^14^ reduced brain volumes,^15^ and impaired brain growth.^16^ There is evidence that reduced total brain volume as well as basal ganglia and brainstem volumes after surgery are associated with impaired cognitive outcomes in infants with CHD.^15^, ^17^

Surgical and clinical risk factors for impaired brain growth include increased length of hospital stay after surgery,^15^, ^18^ older age at surgery,^19^ and underlying cardiac diagnosis.^16^ However, the relationship between perioperative intensive care factors and perioperative brain growth has yet to be fully characterized.

Identifying risk factors for impaired brain growth is crucial to support optimal brain development and to improve neurodevelopmental outcomes for infants born with CHD; it is also necessary to guide factors related to surgical and interventional procedures. Notably, all studies to date investigating perioperative brain growth in infants with CHD have been performed at the group level, which assumes a homogenous effect and does not allow growth trajectories to be assessed at an individual level. As we move toward a more individualized approach in medicine, it is important to identify clinical factors that might impact individual infants with CHD.

Normative neonatal MRI data from the developing Human Connectome Project (dHCP) have previously been used to derive typical trajectories for global and regional brain volumes in neonates between 37 and 44 weeks postmenstrual age.^20^ By mapping individual data sets from infants with CHD to normative data, it is possible to calculate a *Z*‐score that quantifies the degree to which each infant deviates from the typical population at a point in time, in a process akin to growth charts. More important, this method does not assume homogeneous patterns of brain development in individual infants with CHD. We have used this approach to investigate individualized brain volumes in infants with CHD before surgery.^5^ However, to date, it has not been used to investigate perioperative brain growth. By calculating *Z*‐scores for infants with CHD imaged before and after surgery, and calculating their difference across time points, it is possible to investigate individualized regional brain growth trajectories in the perioperative period and to characterize surgical and clinical risk factors associated with impaired perioperative brain growth in this at‐risk cohort.

We aimed to (1) calculate the change in preoperative to postoperative volumetric *Z*‐scores, representing perioperative regional brain volume growth trajectory, for infants with CHD undergoing cardiac surgery or cardiac intervention; and (2) characterize the relationship between change in *Z*‐scores and surgical and clinical risk factors.

## METHODS

The data that support the findings of this study are available from the corresponding author upon reasonable request.

### Recruitment and CHD Categorization

Infants with CHD were prospectively recruited from the Neonatal and Paediatric Intensive Care Units at the Evelina London Children's Hospital (Guy's and St. Thomas' National Health Service Trust), between 2015 and 2022.

Participants were allocated to a category of critical or serious CHD based on previously published classifications.^21^ Critical CHD was defined as infants with hypoplastic left heart syndrome, interrupted aortic arch, pulmonary atresia with an intact ventricular septum, and simple transposition of the great arteries; and all infants requiring surgery or cardiac catheterization within the first 28 days of life with any of the following: aortic valve stenosis, coarctation of the aorta, pulmonary valve stenosis, pulmonary atresia with ventricular septal defect, tetralogy of Fallot, and total anomalous pulmonary venous drainage. Serious CHD was defined as any cardiac lesion not defined as critical, requiring cardiac catheterization or surgery between 1 month and 1 year of age.

Each infant was also allocated to 1 of 3 diagnostic CHD categories based on the hemodynamic impact of the underlying cardiac diagnosis, using the sequential segmental approach^22^: (1) abnormal streaming of blood; (2) left‐sided heart lesions; or (3) right‐sided heart lesions.

### Inclusion Criteria

Infants were eligible for inclusion in this analysis if they had a diagnosis of critical or severe CHD; if they did not undergo any other neonatal surgery before their cardiac surgery or intervention; if preoperative imaging was performed after 37 weeks’ postmenstrual age; and if postoperative imaging was performed before 46 weeks’ postmenstrual age.

### Clinical Data

Clinical data were extracted from each infant's electronic or paper health records, including the following: basic demographic data, postnatal age at surgery, time on cardiopulmonary bypass, circulatory arrest time, length of stay on pediatric intensive care unit (PICU) postoperatively, the need for and duration of any postoperative respiratory support, renal replacement therapy, extracorporeal membrane oxygenation and inotropic support, whether the infant was treated for necrotizing enterocolitis (either medically or surgically), and preoperative serum creatinine levels closest to surgery.

### Magnetic Resonance Imaging

MRI was performed on a Philips Achieva 3‐T system situated on the Neonatal Unit at St Thomas' Hospital (London, UK). Imaging was performed during natural sleep without sedation. Pulse oximetry, respiratory rate, temperature, and electrocardiography were monitored throughout by a nurse and pediatrician experienced in neonatal MRI procedures. Data were acquired with a dedicated 32‐channel neonatal head coil and neonatal positioning system.^23^ Scans included a 5‐second noise ramp‐up to avoid a startle response. T2‐weighted multislice turbo spin echo scans were acquired in 2 stacks in sagittal and axial planes (repetition time/echo time=12 000/156 milliseconds; flip angle=90°; slice thickness=1.6 mm; slice overlap=0.8 mm; in‐plane resolution: 0.8×0.8 mm; and sensitivity encoding  factor=2.11/2.58 [axial/sagittal]. T2‐weighted volumes were reconstructed using a dedicated algorithm to correct motion and integrate data from both acquired stacks (reconstructed voxel size=0.5 mm^3^).^24^, ^25^ T1‐weighted, diffusion‐weighted, susceptibility‐weighted, venographic, and phase‐contrast quantitative flow MRI were also acquired^26^ but not used in this study.

**Figure 1:**
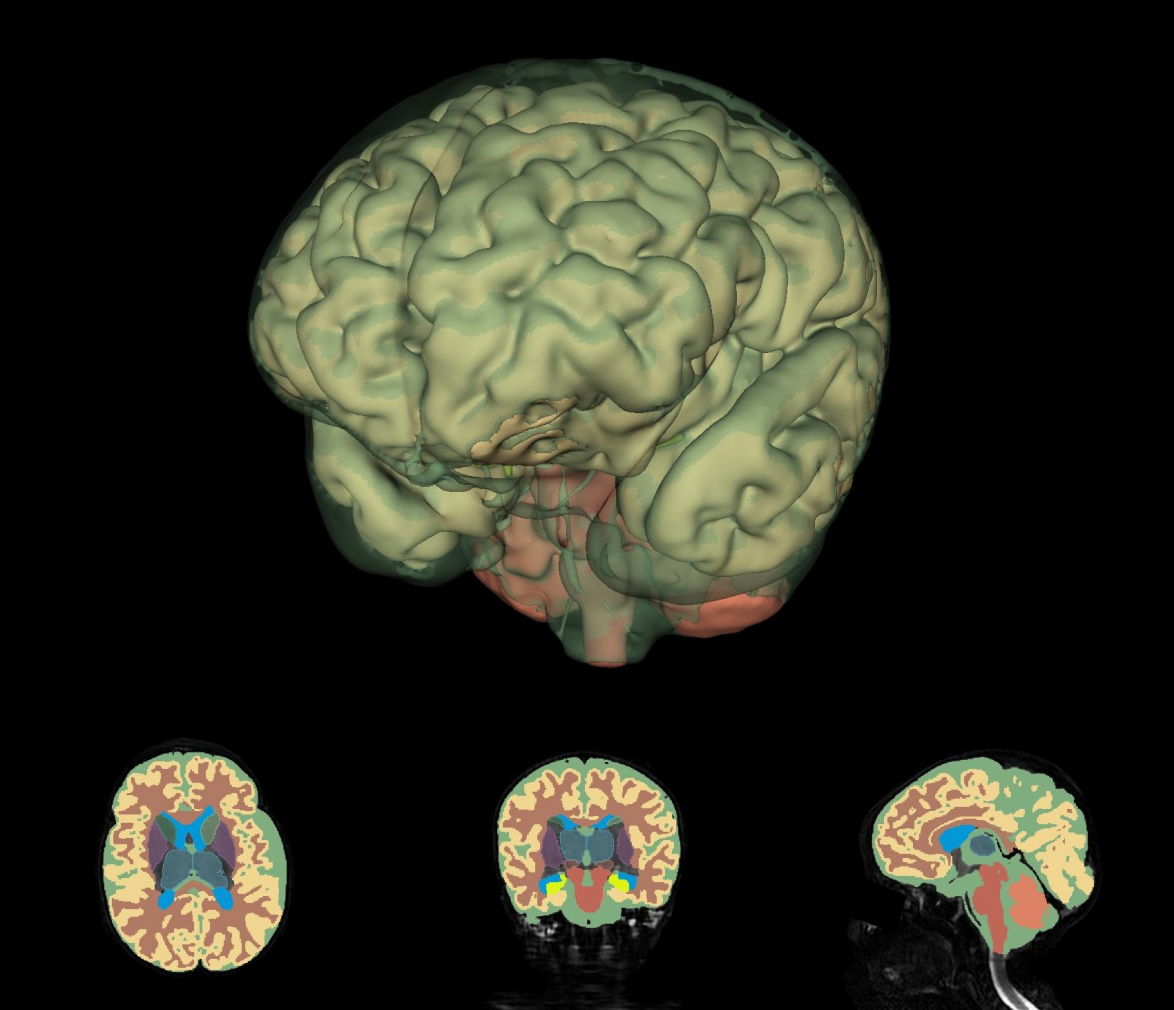
Example 3D reconstruction of an acquired brain MRI image and regional segmentations. A 3D reconstruction (top) of the MRI‐derived regional brain segmentations with CSF (semitransparent), cortex, brainstem, and cerebellum visible. Axial (bottom left), coronal (bottom middle), and sagittal (bottom right) views showing MRI‐derived regional brain segmentations, including CSF (green), cortical gray matter (yellow), white matter (red), ventricles (blue), and deep gray matter structures (caudate nuclei, thalami, subthalamic nuclei, and lentiform nuclei). 3D indicates 3‐dimensional; CSF, cerebrospinal fluid; and MRI, magnetic resonance imaging.

T2‐weighted images were processed using the dHCP structural pipeline.^27^ Images underwent bias correction and brain extraction before being segmented into 8 tissue classes: cortical gray matter, white matter, total deep gray matter, cerebellum, brainstem, hippocampus and amygdala, ventricles, and extracerebral cerebrospinal fluid (CSF), with an automatic neonatal‐specific segmentation algorithm.^28^ Deep gray matter was further segmented into the left and right lentiform and caudate nuclei and thalamus. Segmentations were visually inspected, and minor inaccuracies were manually corrected. Regional tissue volumes were calculated using ITKSnap.^29^ Total tissue volume was calculated by summing cortical gray matter, white matter, cerebellum, brainstem, total deep gray matter, and hippocampus and amygdala (Figure 1).

### MRI Review

All MRI images were reported by experienced perinatal neuroradiologists. The presence of white matter injury, arterial ischemic stroke, cerebellar hemorrhage, cerebral sinus venous thrombosis, intraparenchymal hemorrhage, and ventriculomegaly was recorded.

One infant with CHD had an arterial ischemic stroke in the left parietal lobe that extended into the left postcentral gyrus on both presurgical and postsurgical imaging. Another infant had a large arterial ischemic stroke in the right posterior occipital cortex on postsurgical imaging, affecting cortical gray matter and white matter segmentation accuracy. For these infants, cortical gray matter, white matter volume, and total tissue volume were excluded from further analysis; however, deep gray matter and infratentorial volumes were retained. Ventricular volumes were also excluded from the postsurgical images of the infant with the right occipital arterial ischemic stroke.

### Modeling Typical Development

Typical brain development was modeled using data acquired for the dHCP from a total of 219 healthy infants born after 37 weeks’ gestational age (median [interquartile range] age at birth, 40.1 [39.1–41] gestational weeks; 109 male infants), as described previously.^5^, ^20^

Briefly, Gaussian process regression is a Bayesian nonparametric regression method that has previously been used to model typical volumetric brain development from 37 to 45 weeks’ postmenstrual age^20^ (https://github.com/ralidimitrova/GPR_NeoVols). Gaussian process regression was used to generate separate normative curves for total tissue volume, cortical gray matter, white matter, cerebellum, brainstem, extracerebral CSF, ventricles, left/right caudate, left/right lentiform, and left/right thalamus. Gaussian process regression estimates individual values for each infant and measures the confidence for each prediction. The difference between predicted and observed values normalized by the predictive confidence represents the deviation of a measurement from the expected mean given the infant's postmenstrual age at scan, days of life, and sex. This provides a *Z*‐score quantifying the degree of atypicality in each regional volume, at each scan time point, for each infant with CHD. Changes in brain growth can be represented by the change in slope of *Z*‐scores, where a negative slope when calculating the difference between *Z*‐scores at preoperative and postoperative scans represents a slowing in growth in the perioperative period, a positive slope represents an acceleration in growth, and no change in slope represents consistent growth.

### Statistical Analysis

Extreme deviations from normative volumetric development at each MRI scan were taken as a *Z*‐score of >±2.6 (corresponding to *P*<0.005), representing the top and bottom 0.5% of the typical population.^5^, ^20^ Significant changes in *Z*‐score slopes were examined at 3 thresholds: >±2.6 (corresponding to *P*<0.005), >±2.3 (corresponding to *P*<0.01), and >±1.65 (corresponding to *P*<0.05). Infants with extreme deviations were examined for cardiac diagnosis, gestational age at birth, sex, and brain injury.

The Fisher exact test was used to compare the proportion of deviations across the type of CHD (abnormal streaming of blood, left‐sided heart lesions, and right‐sided heart lesions) and clinical categorical variables.

The Shapiro‐Wilk test was used to test normality. The Wilcoxon ranked sum test was used to compare *Z*‐scores before and after surgery. The Kruskal‐Wallis 1‐way test of variance was used to compare the change in *Z*‐score slope between categories of CHD. Spearman rank correlations were used to characterize the relationship between change in *Z*‐score slope and postnatal age at surgery, time between surgery and postoperative MRI, and time between preoperative and postoperative MRI. Partial Spearman rank correlations were used to characterize the relationship between change in *Z*‐score slope and days on PICU postoperatively, including age at surgery as a covariate. Partial Spearman rank correlations were used to characterize the relationship between change in *Z*‐score slope and duration of bypass and circulatory arrest time, including days on PICU postoperatively as a covariate.

To determine if the relationship between change in *Z*‐score slope was related to impaired somatic growth, infant weight and head circumference were measured at each scan and converted to *Z*‐scores using the UK–World Health Organization growth centiles.^30^ Spearman rank correlations were used to test the association between change in head circumference and scan weight *Z*‐scores and change in volumetric *Z*‐score slopes, age at surgery, time on bypass, circulatory arrest time, and days on PICU.

Mann‐Whitney *U* and Spearman rank correlations were used to test the relationship between change in volumetric *Z*‐score slopes and renal replacement therapy, necrotizing enterocolitis, duration of ventilation postoperatively, and duration of inotrope treatment postoperatively. A post hoc analysis was undertaken using partial Spearman rank correlation, to assess the association between preoperative serum creatinine levels and perioperative brain growth, with postnatal age at creatinine measurement as a covariate.

Benjamini and Hochberg false discovery rate (FDR) was applied to correct for multiple comparisons (reported as *P*_FDR_). All statistical analyses were performed in R v3.6.2.

## RESULTS

Our prospective cohort included 36 infants with critical or serious CHD (19 male infants; median [interquartile range] gestational age at birth, 38.5 [38.1–39.0] weeks). Table 1 provides full demographics.

**Table 1:** Summary of Cohort Demographic Information

Demographic data	Value (N=36)
Gestational age at birth, median (IQR), wk	38.5 (38.1 to 39.0)
Postmenstrual age at preoperative scan, median (IQR), wk	39.4 (38.7 to 39.9)
Postmenstrual age at postoperative scan, median (IQR), wk	41.9 (41.0 to 42.7)
CHD diagnosed antenatally, N (%)	34 (94)
Birth weight, mean±SD, g	3146±572
Head circumference, mean±SD, cm	33.8±1.77
Postnatal age at preoperative scan, median (IQR), d	5 (2.75 to 7.0)
Postnatal age at postoperative scan, median (IQR), d	21.5 (15 to 29.75)
Postnatal age at cardiac surgery/intervention, median (IQR), d	12 (7 to 14.25)
Weight at preoperative scan *Z*‐score, median (IQR)	0.15 (−1.11 to 0.81)
Weight at postoperative scan *Z*‐score, median (IQR)	−0.99 (−2.30 to −0.17)
Head circumference at preoperative scan *Z*‐score, median (IQR)	0.31 (−0.50 to 0.82)
Head circumference at postoperative scan *Z*‐score, median (IQR)	−0.59 (−1.58 to 0.27)
Time between intervention and postoperative MRI, median (IQR), d	8 (6.75 to 11.25)
Time between preoperative and postoperative MRI, median (IQR), d	14 (10.75 to 22.00)
Primary cardiac defect, N (%)	
Abnormal streaming of blood	20 (56)
Transposition of the great arteries	*17* (*47*)
Total anomalous pulmonary venous drainage	*1* (*3*)
Truncus arteriosus	*2* (*6*)
Left‐sided cardiac lesions	12 (33)
Aortic stenosis	*1* (*3*)
Coarctation of the aorta	*10* (*28*)
Hypoplastic aortic arch	*1* (*3*)
Right‐sided cardiac lesions	4 (11)
Pulmonary atresia	*3* (*8*)
Pulmonary stenosis	*1* (*3*)
Delivery method, N (%)	
Spontaneous/induced vaginal delivery	18 (50)
Instrumental delivery	3 (8)
Elective cesarean section	7 (19)
Emergency cesarean section	8 (22)
Surgical and clinical factors	
Cardiac intervention by catheterization, N (%)*	3 (8)
Cardiopulmonary bypass, N (%)	31 (86)
Time on bypass, median (IQR), min	153 (128 to 162)
Time on intensive care after intervention, median (IQR), d	4 (3 to 5)
Renal replacement therapy, N (%)	6 (17)
Extracorporeal membrane oxygenation, N (%)	0 (0)
Necrotizing enterocolitis, N (%)	4 (11)
Time on ventilation after intervention, median (IQR), d	3 (2 to 4)
Time on inotropes after intervention, median (IQR), d	3 (2 to 4)

CHD indicates congenital heart disease; IQR, interquartile range; and MRI, magnetic resonance imaging.

* Excluding balloon atrial septostomy (N=9).

Italics values indicate the transposition of the great arteries, total anomalous pulmonary venous drainage and truncus arteriosus are types lesions causing of abnormal streaming of blood etc.

### Changes in Brain Growth in the Perioperative Period

**Figure 2:**
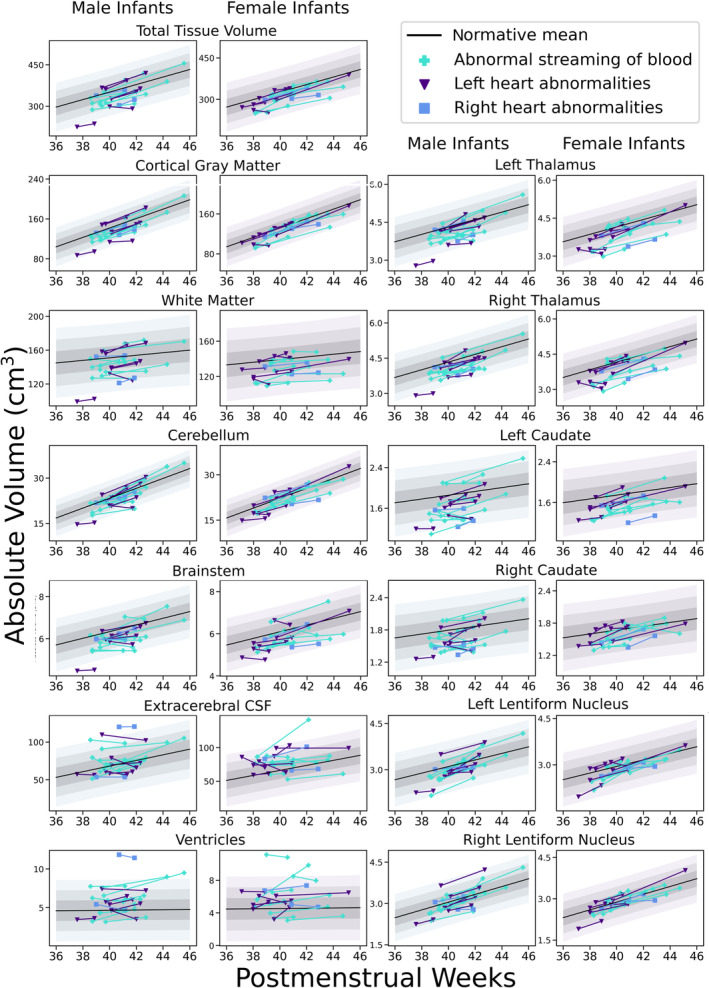
Preoperative and postoperative volumetric brain development in neonates with CHD. Plots showing preoperative and postoperative volumetric brain development in neonates with CHD. Shaded areas represent 1, 2, and 3 SDs from the normative model mean. Results are shown separately for female and male infants. CHD indicates congenital heart disease; CSF, cerebrospinal fluid.

*Z*‐scores were significantly more negative after surgery compared with before surgery in cortical gray matter, white matter, cerebellum, brainstem, right thalamus, right lentiform, total tissue volume, and extracerebral CSF spaces (Table 2 and Figure 2). There was no significant change in *Z*‐scores in the ventricles, left thalamus, left lentiform, or caudate nuclei. Changes in *Z*‐score slopes were not associated with the category of CHD (all *P*_FDR_>0.315; Table S1).

**Table 2:** Preoperative and Postoperative Volumetric *Z*‐Scores in Infants With CHD

Region	Preoperative *Z*‐score, median (IQR)	Postoperative *Z*‐score, median (IQR)	*Z*‐score slope change, median (IQR)	*P*_FDR_
Extracerebral CSF	0.804 (−0.172 to −1.732)	0.042 (−0.502 to −0.931)	−0.498 (−0.896 to −0.220)	0.001*
Cortical gray matter	−0.631 (−1.155 to 0.010)	−0.857 (−1.696 to −0.217)	−0.303 (−0.507 to −0.112)	<0.001*
White matter	−0.827 (−1.379 to −0.027)	−0.726 (−1.692 to −0.194)	−0.095 (−0.455 to 0.074)	0.049*
Ventricles	0.441 (−0.096 to 1.495)	0.658 (−0.114 to 1.624)	0.071 (−0.346 to 0.424)	0.566
Cerebellum	−0.478 (−1.20 to 0.163)	−1.144 (−1.860 to 0.145)	−0.661 (−1.251 to −0.085)	<0.001*
Brainstem	−0.873 (−1.368 to −0.075)	−1.657 (−2.303 to −0.518)	−0.751 (−1.119 to −0.127)	<0.001*
Left thalamus	−0.628 (−1.622 to −0.275)	−0.986 (−1.914 to −0.231)	−0.093 (−0.552 to 0.176)	0.363
Right thalamus	−0.858 (−1.697 to −0.187)	−0.994 (−2.056 to −0.480)	−0.263 (−0.641 to 0.183)	0.037*
Left caudate	−1.157 (−1.822 to −0.661)	−1.171 (−1.967 to −0.629)	0.026 (−0.258 to 0.295)	0.932
Right caudate	−0.873 (−1.368 to −0.027)	−0.742 (−1.565 to −0.147)	−0.070 (−0.276 to 0.259)	0.458
Left lentiform	−0.684 (−1.060 to −0.016)	−0.512 (−0.799 to 0.241)	0.313 (−0.059 to 0.583)	0.132
Right lentiform	−0.339 (−0.724 to 0.304)	−0.446 (−0.909 to 0.231)	−0.151 (−0.508 to 0.169)	0.014*
Total tissue volume	−0.800 (−1.310 to −0.049)	−0.864 (−1.919 to −0.354)	−0.220 (−0.537 to 1.114)	<0.001*

CHD indicates congenital heart disease; CSF, cerebrospinal fluid; FDR, false discovery rate; and IQR, interquartile range.

* Significant results.

#### Significant Deviations in Brain Development

Extreme deviations in *Z*‐scores and significant changes in *Z*‐score slopes in infants with CHD are summarized in Table S2. Individuals with deviations are summarized in Table S3.

Significant changes in *Z*‐score slopes were present in 1 infant (2.8%; abnormal streaming, N=1) at a threshold of ±2.6 (*P*=0.005); 2 infants (16.7%; abnormal streaming, N=2) at a threshold of ±2.3 (*P*=0.01); and 13 infants (36.1%; abnormal streaming, N=8; left sided, N=5) at a threshold of ±1.65 (*P*=0.05).

When examining infants with significant changes in *Z*‐score slopes (threshold: ±1.65; N=13), 4 infants showed isolated normalization of extracerebral CSF slopes toward the normative mean (median *Z*_pre_=2.11; median *Z*_post_=0.259). One infant had significantly increased right lentiform slope (*Z*_pre_=−0.326; *Z*_post_=1.53). One infant had significantly increased right lentiform slope as well as extracerebral CSF and ventricle expansion. The remaining 7 infants showed negative slopes of at least 1 regional tissue volume. CHD group was unrelated to extreme deviations before or after surgery, or deviations in brain growth (Table S4).

### Associations Between Surgery and Timing of MRI With Changes in Brain Growth

**Figure 3:**
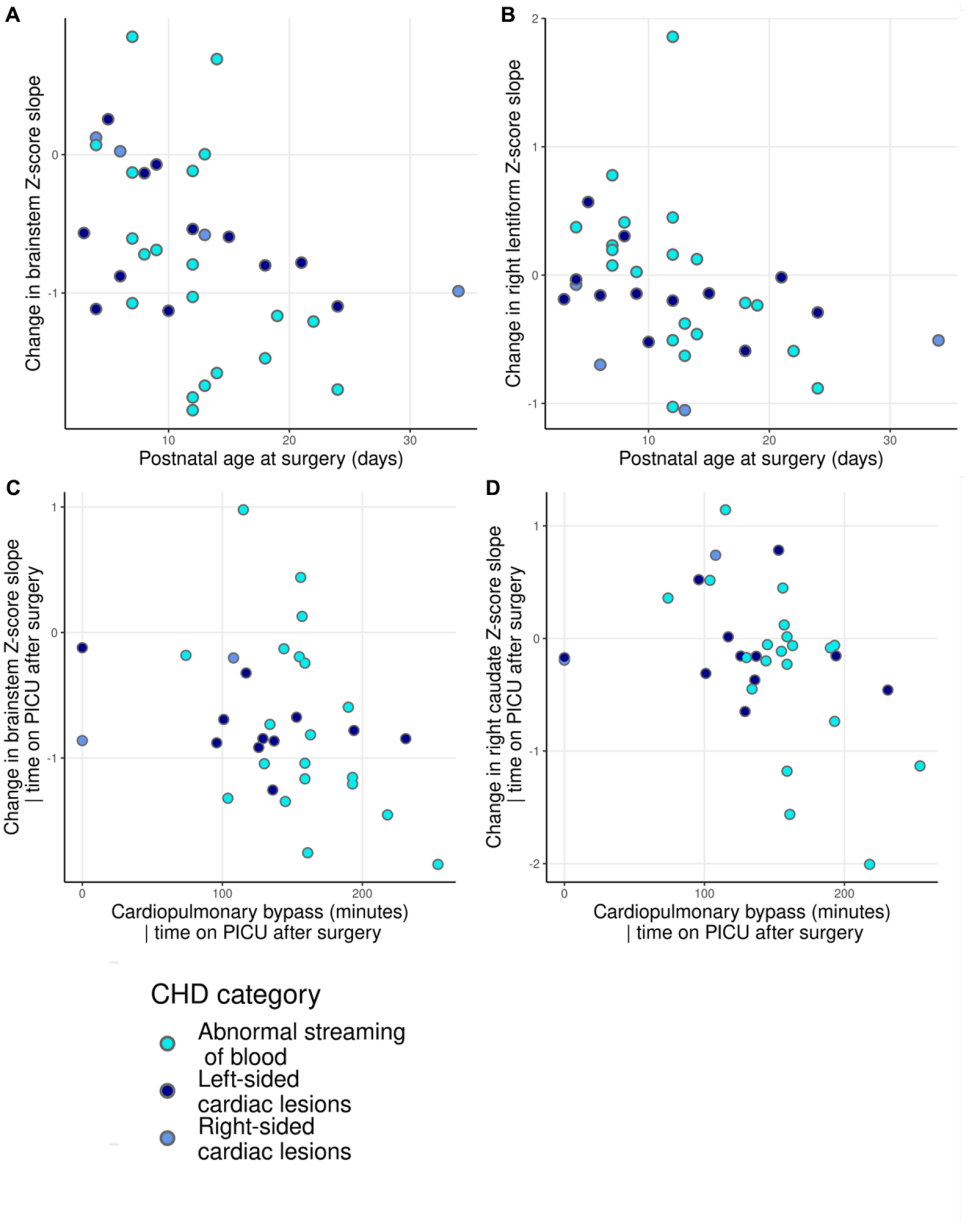
Association between age at surgery and change in regional brain volume slopes preoperatively to postoperatively. Plots to show the association between age at surgery and change in regional volume slope preoperatively to postoperatively in brainstem (**A**) and right caudate nucleus (**B**); and the relationship between duration of cardiopulmonary bypass and change in slope in brainstem (**C**) and right caudate nucleus (**D**), correcting for time on PICU postoperatively. CHD indicates congenital heart disease; PICU, pediatric intensive care unit.

Older postnatal age at surgery was associated with increased negative slope in *Z*‐scores in the brainstem (⍴=−0.481; *P*_FDR_=0.042) and right lentiform (⍴=−0.477; *P*_FDR_=0.042) (Table S5 and Figure 3). Time between preoperative and postoperative MRI and timing of postoperative MRI were not related to change in *Z*‐score slopes in any region (⍴<0.325; *P*>0.173) (Table S5). Changes in weight and head circumference *Z*‐score between scans were not correlated with age at surgery (weight ⍴=−0.096, *P*_FDR_=0.578; head circumference ⍴=−0.154, *P*_FDR_=0.567; Table S6).

### Duration of Postoperative PICU Stay Is Associated With Changes in Brain Growth

**Figure 4:**
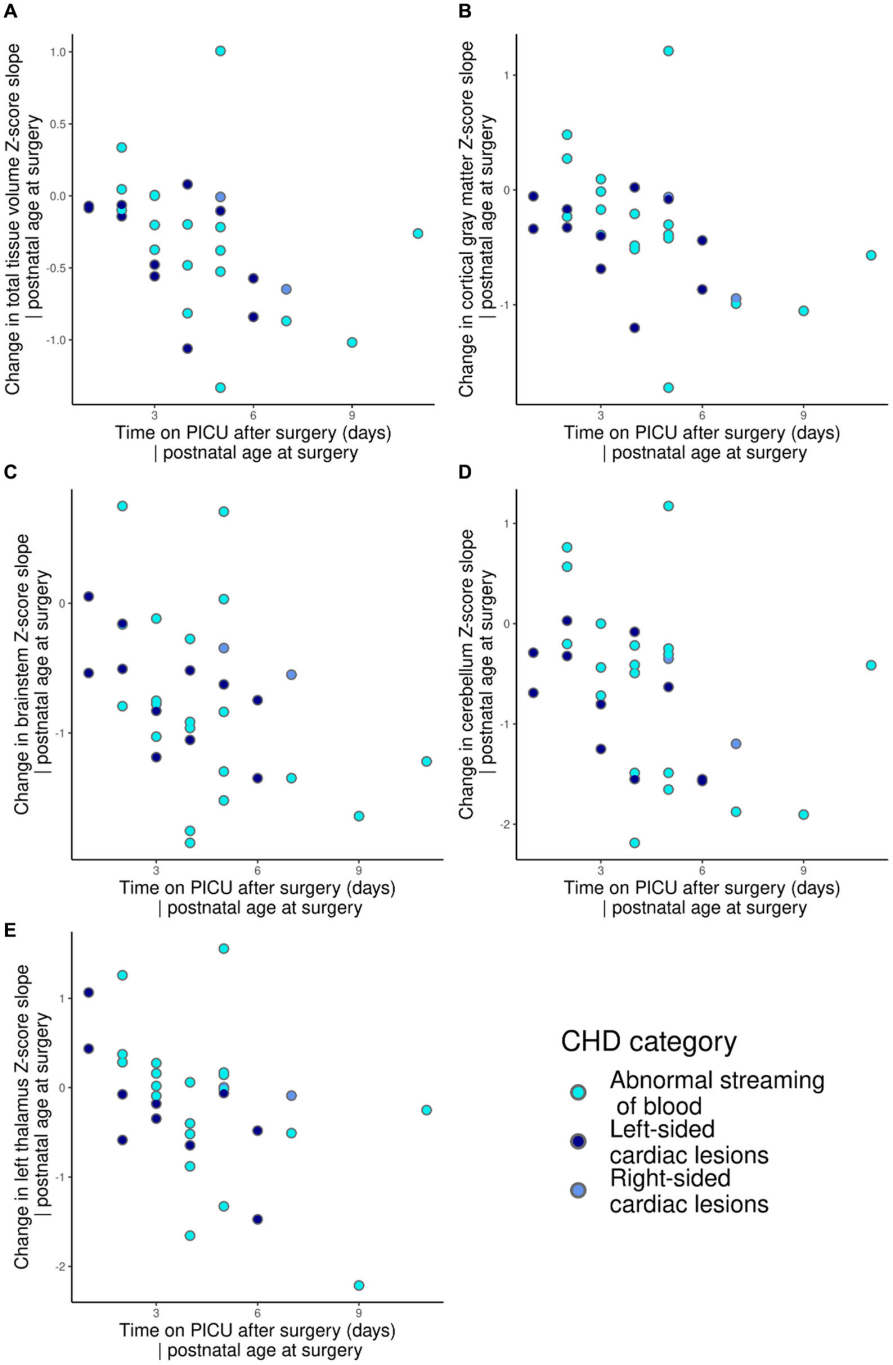
Relationship between time on PICU postoperatively and change in regional brain volume slopes preoperatively to postoperatively. Plots to show the relationship between time on PICU postoperatively and change in regional volume slope preoperatively to postoperatively for total tissue volume (**A**), cortical gray matter (**B**), brainstem (**C**), cerebellum (**D**), and left thalamus (**E**). CHD indicates congenital heart disease; and PICU, pediatric intensive care unit.

Longer duration of PICU stay postoperatively was associated with larger negative slopes of *Z*‐scores in cortical gray matter, white matter, cerebellum, brainstem, left thalamus, and total tissue volume, when correcting for age at surgery (Table 3 and Figure 4). Duration of PICU stay was not associated with change in weight (⍴=−0.355; *P*_FDR_=0.156) or head circumference (⍴=−0.349; *P*_FDR_=0.151) *Z*‐score.

**Table 3:** Relationship Between *Z*‐Score Slopes and Duration of PICU Stay, Covarying for Age at Surgery

*Z*‐score slope	ρ	*P*_FDR_
Extracerebral CSF	0.022	0.977
Cortical gray matter	−0.507*	0.015*
White matter	−0.349	0.086
Ventricles	0.002	0.990
Cerebellum	−0.495*	0.015*
Brainstem	−0.419*	0.039*
Right caudate	−0.214	0.336
Left caudate	−0.134	0.593
Right lentiform	−0.338	0.089
Left lentiform	−0.097	0.698
Right thalamus	−0.391	0.053
Left thalamus	−0.456*	0.027*
Total tissue volume	−0.590*	0.004*

CSF indicates cerebrospinal fluid; FDR, false discovery rate; and PICU, pediatric intensive care unit.

* Results are significant.

### Individual Clinical Course Is Associated With Reduced Brain Growth

**Figure 5:**
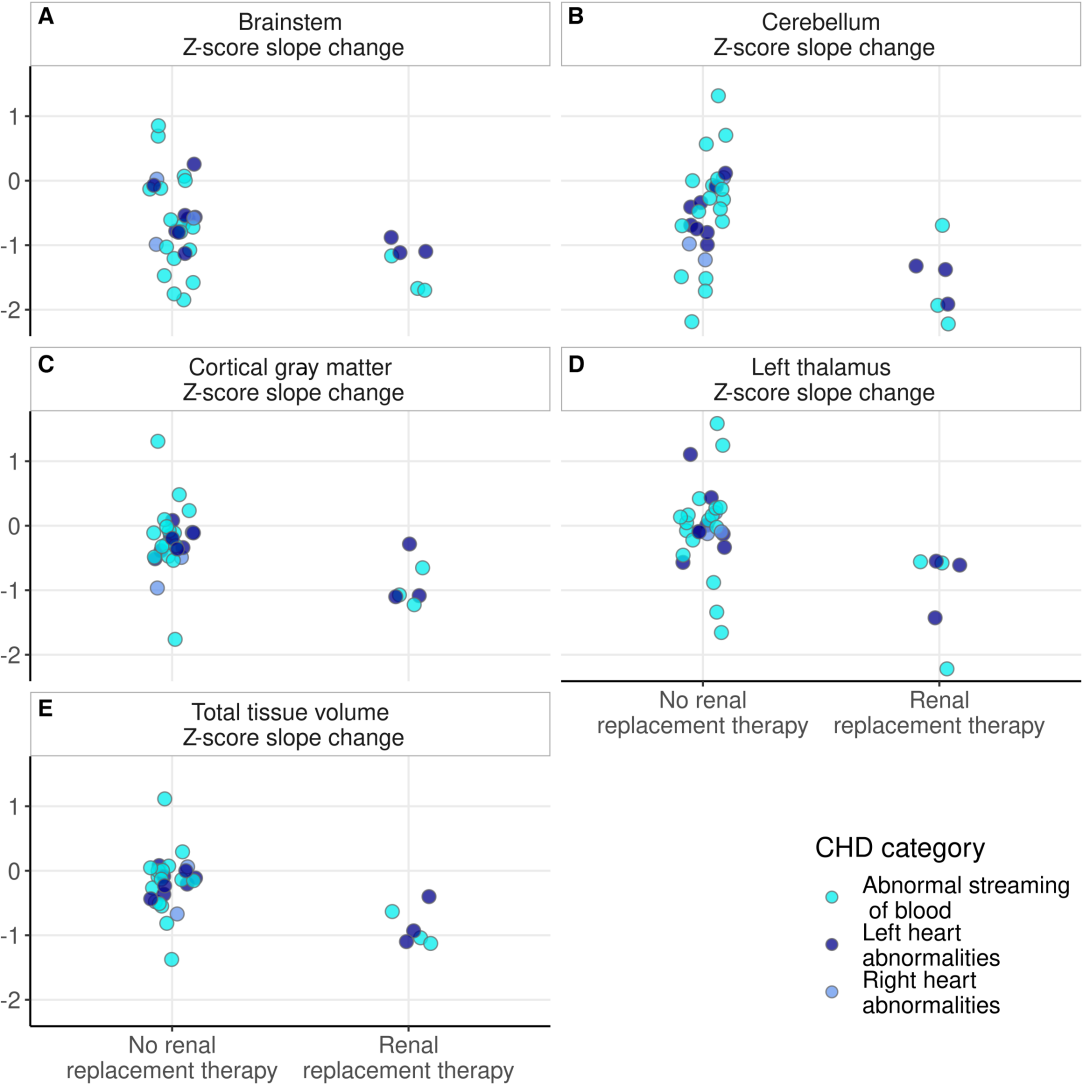
Relationship between postoperative renal replacement therapy and *Z*‐score slope change in cortical gray matter, cerebellum, and left thalamus volumes. Plots to show the *Z*‐score slope change preoperatively to postoperatively in brainstem (**A**), cerebellum (**B**), cortical gray matter (**C**), left thalamus (**D**), and total brain tissue volume (**E**) for infants grouped according to the need for postoperative renal replacement therapy. CHD indicates congenital heart disease.

**Figure 6:**
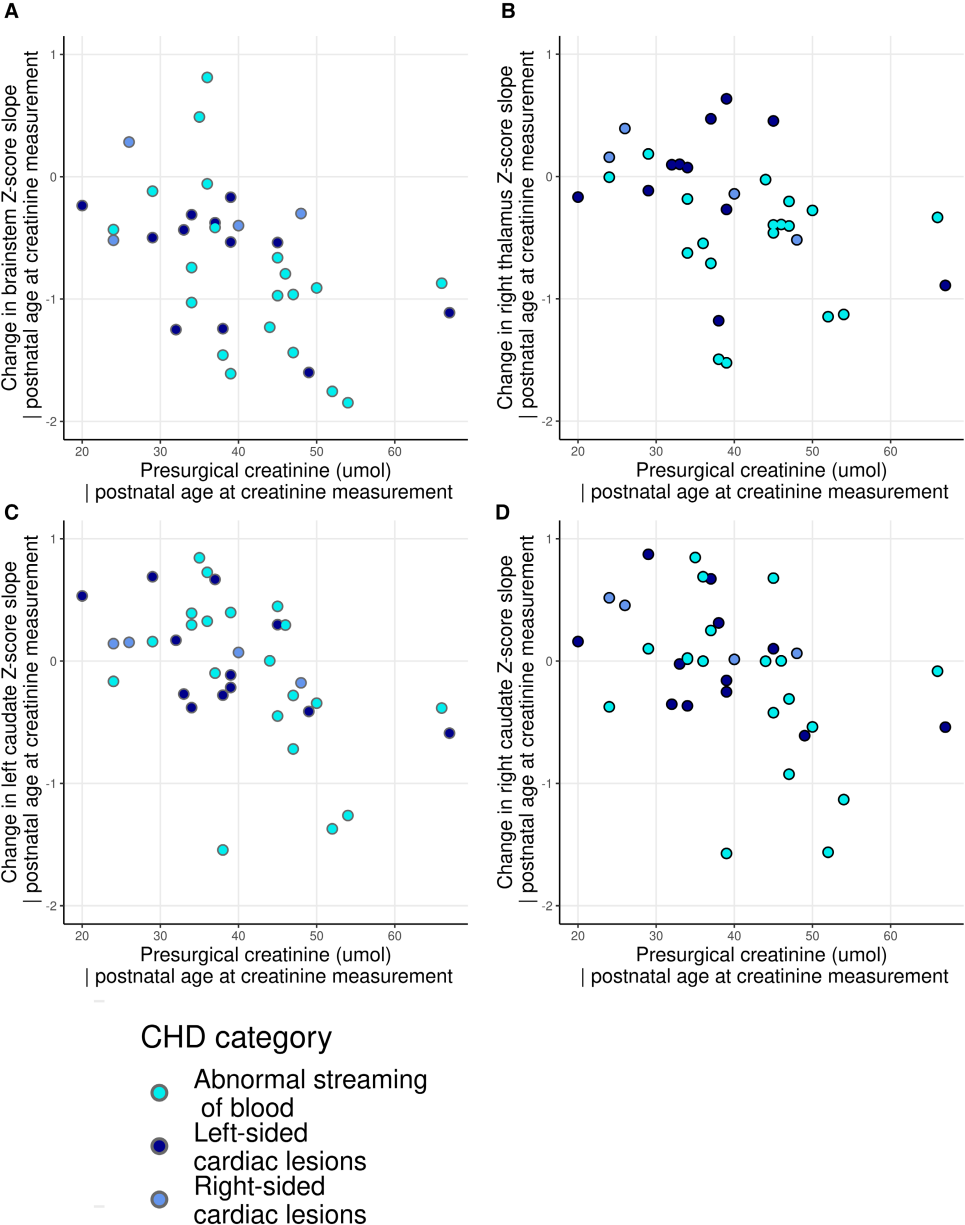
Relationship between preoperative serum creatinine levels and change in regional brain volume slopes preoperatively to postoperatively. Plots to show the relationship between preoperative serum creatinine and change in regional volume slope preoperatively to postoperatively for brainstem (**A**), right thalamus (**B**), left caudate (**C**), and right caudate (**D**). CHD indicates congenital heart disease.

Post hoc analyses revealed that postoperative renal replacement therapy was associated with larger negative slopes in *Z*‐scores in cortical gray matter, cerebellum, and left thalamus (Table 4 and Figure 5). Change in left thalamus slope was also associated with duration of inotrope treatment after surgery. A total of 86% (N=6) of infants with significant negative slopes in brain tissue underwent renal replacement therapy or were diagnosed with necrotizing enterocolitis postoperatively compared with 14% (N=4) without (*P*<0.001). Higher preoperative serum creatinine levels (median [interquartile range], 39 [34–46] μmol/L) were significantly associated with larger negative *Z*‐score slopes in the brainstem (⍴=−0.503; *P*_FDR_=0.033), caudate nuclei (left ⍴=−0.476, *P*_FDR_=0.033; right ⍴=−0.434, *P*_FDR_=0.033), and right thalamus (⍴=−0.436; *P*_FDR_=0.038) when covarying for postnatal age at creatinine measurement (Table S7 and Figure 6). Preoperative serum creatinine levels were not related to duration of postoperative PICU stay (⍴=−0.065; *P*=0.720) or need for postoperative renal replacement therapy (U=80.5; *P*=0.892).

**Table 4:** Post Hoc Relationship Between Intensive Care Factors and *Z*‐Score Slope

Region	Renal replacement therapy		Necrotizing enterocolitis		Duration of invasive ventilation		Duration of inotropic support	
	Median difference	*P*_FDR_	Median difference	*P*_FDR_	ρ	*P*_FDR_	ρ	*P*_FDR_
Cortical gray matter	0.737*	0.024*	0.735	0.110	−0.276	0.148	−0.221	0.110
Cerebellum	1.19*	0.024*	0.935	0.072	−0.370	0.072	−0.303	0.072
Brainstem	0.574	0.052	0.631	0.105	−0.193	0.288	−0.217	0.072
Left thalamus	0.764*	0.022*	1.057	0.072	−0.360	0.072	−0.394*	0.028*
Total tissue volume	0.697*	0.022*	0.598	0.110	−0.300	0.119	−0.217	0.105

FDR indicates false discovery rate.

* Results are significant.

### Preoperative or Postoperative Brain Injury Is Not Associated With White Matter Volume Changes

Preoperative brain injury was identified in 17 infants (12 with white matter injury), postoperative injury was identified in 24 infants (18 with white matter injury), and new postoperative injury was identified in 17 infants (14 with new white matter injury). Brain injuries in individual infants are described in Table S8. There were no associations between presence or absence of preoperative, postoperative, or new postoperative brain injury or white matter injury and change in *Z*‐score slope (Table S9).

### Associations Between Intraoperative Variables and Change in Brain Growth

Increased negative slopes in *Z*‐scores in the brainstem (⍴=−0.518; *P*_FDR_=0.026) and right caudate nucleus (⍴=−0.587; *P*_FDR_=0.009) were associated with longer time on cardiopulmonary bypass, after correcting for time on PICU after surgery (Table S10 and Figure 3). Cardiopulmonary bypass time was not related to change in weight (⍴=−0.298; *P*_FDR_=0.117) or head circumference (⍴=−0.116; *P*_FDR_=0.578) *Z*‐score. There were no significant associations between *Z*‐score slopes in any regional volumes and circulatory arrest time during surgery (all ⍴ < 0.46; *P*_FDR_>0.061).

Changes in preoperative to postoperative head circumference and weight *Z*‐scores were not associated with change in volumetric *Z*‐score slopes (Table S6).

## DISCUSSION

This study used normative modeling techniques to investigate individualized brain growth in the perioperative period and explore risk factors for impaired perioperative brain development in infants with CHD. Growth in cortical gray matter, white matter, cerebellum, brainstem, right lentiform nucleus, right thalamus, and total tissue volume was significantly impaired in the immediate postoperative period. However, extracerebral CSF volume *Z*‐scores were significantly reduced after surgery, with absolute postoperative CSF volumes being close to the typical mean. Brainstem growth was particularly vulnerable to clinical course, with preoperative creatinine levels, later age at surgery, longer duration of cardiopulmonary bypass, and longer time on intensive care postoperatively associated with impaired growth in this region. Longer time on intensive care postoperatively was also associated with impaired growth of cortical gray matter, cerebellum, left thalamus, and total tissue volume. Impaired growth in the right lentiform and right caudate nuclei was associated with later age at surgery and longer time on cardiopulmonary bypass, respectively. Significant negative deviations in perioperative intraparenchymal regional brain growth were identified in 19% of infants in the study.

To our knowledge, this is the first use of a normative modeling approach to assess perioperative brain development in infants with CHD to identify significant changes in individual brain growth trajectories. Our results demonstrate an overall slowing of intracranial growth in the immediate postoperative period. Although group studies have reported larger differences in brain tissue volumes between infants with CHD and controls after surgery compared with before,^15^, ^31^ our study highlights the heterogeneity of perioperative trajectories in infants with CHD. These range from extremely impaired to stable or even accelerated growth in regional intracranial volumes.

Although *Z*‐scores showed extracerebral CSF volumes were enlarged before surgery, in keeping with our previous findings,^5^ overall postoperative extracerebral CSF volumes were close to the typical mean. In a cohort of infants with CHD composed of cross‐sectional and longitudinal data, Claessens and colleagues^17^ demonstrated that extracerebral CSF volumes did not expand at the same rate as intraparenchymal tissue volumes in the perioperative period. Similarly, Ortinau and colleagues^31^ reported CSF volumes in infants with CHD, >40% of whom had hypoplastic left heart syndrome, were not different from healthy controls in the immediate perioperative period; however, they were significantly enlarged at 3 months of age. Indeed, Heye and colleagues^32^ identified enlarged CSF spaces in toddlers with single‐ventricle cardiac physiological features before Fontan completion. It is unclear whether these different findings in extracerebral CSF volumes are related to differences in surgical management or underlying cardiac physiological features. It is possible that repair of cardiac defects may contribute to normalizing extracerebral CSF volumetric growth in the immediate postoperative period; however, this requires further investigation.

Later postnatal age at surgery was associated with significantly impaired perioperative growth of the brainstem and right lentiform nucleus. Later surgery has been linked to lower estimated brain weight *Z*‐scores and impaired early language abilities in infants with transposition of the great arteries,^33^ and with poorer clinical outcomes in infants with hypoplastic left heart syndrome.^34^ Ibuki and colleagues report accelerated whole brain growth between 1 and 3 years of age in infants with transposition of the great arteries who undergo corrective surgery but not single‐ventricle physiological features, who are operated palliatively, and that normalization of cerebral oxygenation by corrective surgery was associated with improved brain growth.^35^ Impaired cerebral oxygen delivery is related to the degree of brain volume reduction before surgery in infants with CHD.^5^, ^8^ It is possible that prolonged exposure to impaired cerebral oxygen delivery may constrain postnatal brainstem and right lentiform growth in infants with CHD.

Impaired growth of the brainstem and right caudate nucleus was associated with longer time on cardiopulmonary bypass independent of postoperative intensive care stay. Longer time on cardiopulmonary bypass has previously been linked to more severe or new white matter injury postoperatively^13^ and greater neurodevelopmental impairment in early childhood.^36^ Cortical gray matter and white matter development is impaired in juvenile porcine models of congenital cardiac surgery.^37^, ^38^ However, to our knowledge, no study has investigated the impact of cardiopulmonary bypass on subcortical gray matter and infratentorial development. Cardiopulmonary bypass may expose the brain to acute hypoxia/ischemia and reperfusion injury,^39^, ^40^ with the basal ganglia being particularly susceptible to acute severe hypoxic ischemic injury in infancy,^41^, ^42^ in part attributable to higher metabolic requirements. Our findings suggest that the brainstem and basal ganglia nuclei may be susceptible to impaired development associated with longer cardiopulmonary bypass time in infants with CHD.

Duration of postoperative intensive care stay was associated with the degree of impaired perioperative growth of total tissue volume, and cortical gray matter, brainstem, cerebellum, and left thalamus volumes in particular. More important, both duration of intensive care stay and *Z*‐score slopes were unrelated to changes in somatic growth trajectory, suggesting the effect is specific to brain growth. It has previously been reported that a longer hospital stay was associated with impaired total brain growth^15^ and relative growth of the left planum temporale^18^ in infants with CHD. Prolonged postoperative intensive care stay has also been associated with abnormal background activity on amplitude‐integrated electroencephalography^3^ in this population. Interestingly, 86% of the infants with extreme reductions in regional brain growth had a postoperative extracardiac complication, either requiring renal replacement therapy or a diagnosis of necrotizing enterocolitis. These extracardiac complications may reflect prolonged exposure to hypoxia/ischemia throughout the fetal, preoperative, and perioperative periods.^43^, ^44^ Taken together, our results suggest infants requiring prolonged postoperative intensive care, possibly related to complications arising from systemic hypoxia/ischemia, are at highest risk of impaired perioperative brain growth.

Interestingly, higher preoperative serum creatinine levels, despite all being within the (laboratory‐specific) normal ranges, were associated with impaired growth in the brainstem and basal ganglia. As a readily available measure of renal function, serum creatinine may represent a potentially modifiable preoperative clinical biomarker in this population. Strategies to optimize renal function and minimize the risk of acute kidney injury perioperatively, such as strict avoidance of nephrotoxic medicines preoperatively, and close monitoring of fluid balance, may ameliorate impaired growth of the brainstem and the basal ganglia in infants with CHD; however, this hypothesis requires further investigation.

Impaired brainstem growth was associated with preoperative creatinine levels, later age at surgery, longer duration of cardiopulmonary bypass, and longer postoperative time on intensive care, suggesting this region is uniquely sensitive to clinical course. Previous research in infants born prematurely and those exposed to hypoxic/ischemic injury reports associations between impaired brainstem development and longer time on intensive care and extended mechanical ventilation.^45^, ^46^ Similarly, reduced brainstem volumes have been reported in infants who underwent repair for long gap esophageal atresia.^47^ The brainstem is particularly vulnerable to inflammatory, vascular, and excitotoxic insults in critically ill patients.^48^ Indeed, in vitro analyses of rat tissue reveal brainstem neurons are more sensitive to hypoxia than cortical neurons.^49^ Taken together, this suggests the brainstem growth is particularly vulnerable to perinatal illness.

Geva and Feldmann^41^ have proposed that compromised brainstem function is an antecedent of impaired long‐term cognitive and behavioral outcomes in infants exposed to perinatal adversities. They suggest impaired physiological homeostasis as a result of brainstem dysfunction may impair an infant's ability to attend external stimuli, modulate arousal, and react to stress, leading to altered sleep, feeding, and self‐soothing. This may, in turn, disrupt the management of negative emotions and the development of inhibitory control and self‐regulation. Reduced neonatal brainstem volumes have been linked to impaired cognitive and motor outcomes in children with CHD.^17^ We suggest that the brainstem may be susceptible to cumulative exposures before, during, and after the perioperative period, which may mediate the relationship between clinical variables and impaired neurodevelopmental outcomes in CHD; however, this hypothesis requires further investigation.

### Limitations

The normative data needed to robustly model typical brain development were available for infants aged 37 to 46 weeks’ postmenstrual age who were scanned as part of the dHCP. Although most infants with left‐sided cardiac lesions (eg, coarctation of the aorta) or abnormal streaming of blood (eg, transposition of the great arteries) undergo surgery within the first few weeks of life, many infants with right‐sided lesions (eg, those with tetralogy of Fallot) do not typically undergo surgery until several months of age. We were therefore unable to investigate perioperative brain development in these infants, and our sample of infants with right‐sided cardiac lesions was particularly small.

The cohort studied here includes only 1 infant with single‐ventricle physiological features (pulmonary atresia with central aortopulmonary shunt) and no infants with hypoplastic left heart syndrome receiving a Norwood procedure. All other infants with "left‐sided lesions" underwent complete anatomic repair, with 2‐ventricle physiological features. This may explain why the CHD grouping used in this study did not demonstrate any significant between‐group differences for changes in perioperative brain growth.

Postoperative *Z*‐scores in multiple brain regions were significantly more negative compared with preoperative *Z*‐scores. However, all imaging was performed in the first few weeks of life, with postoperative imaging taking place in the immediate postoperative period, typically before discharge. Brain growth in infants with CHD, particularly those undergoing complete surgical repair, may accelerate in later infancy. We, therefore, cannot draw any conclusions about the long‐term trajectories of brain growth of infants with CHD. Future research applying normative methods to brain MRI data from older infants and young children with CHD is needed to understand long‐term brain growth trajectories in this population.

We explored macrostructural brain changes in the perioperative period. Future studies are required to determine the microstructural tissue changes underlying volume changes that are detectable with structural MRI. In this observational study, it was not possible to definitively disentangle how different underlying cardiac physiological features, clinical courses, and brain growth interact.

Finally, we did not assess neurodevelopment in this cohort. Future studies, when these infants are toddlers, will assess the impact of impaired perioperative brain development on later outcomes.

## CONCLUSIONS

Infants with CHD demonstrate normalization of extracerebral CSF volumes while being at increased risk of impaired brain tissue growth in the immediate postoperative period. The brainstem appears to be particularly vulnerable to the perioperative clinical course. Impaired growth of the basal ganglia nuclei is related to longer time to surgery and longer duration of cardiopulmonary bypass, perhaps reflecting a particular vulnerability of these regions to acute and chronic hypoxic/ischemic injury. Longer intensive care stay postoperatively is associated with impaired brain growth. Further research is needed to confirm whether postoperative extracardiac complications related to systemic hypoxia/ischemia are specific risk factors for impaired brain growth in this population and whether alterations in brain growth identified in the immediate postoperative period persist into later childhood and beyond. However, these results provide opportunities to optimize perioperative brain growth in individual neonates with CHD.

## Sources of Funding

This research was funded by the Medical Research Council UK (MR/L011530/1; MR/V002465/1), the British Heart Foundation (FS/15/ 55/31649) and Action Medical Research (GN2630). The normative sample was collected as part of the Developing Human Connectome Project, funded by the European Research Council under the European Union’s Seventh Framework Program (FP7/20072013)/European Research Council grant agreement no. 319456. This research was supported by the Wellcome Engineering and Physical Sciences Research Council Centre for Medical Engineering at King’s College London (WT 203148/Z/16/Z), Medical Research Council UK strategic grant (MR/K006355/1), Medical Research Council UK Centre grant (MR/N026063/1) and by the National Institute for Health Research (NIHR) Biomedical Research Centre based at Guy’s and St Thomas’ NHS Foundation Trust and King’s College London. LCG is supported by the Comunidad de Madrid‐Spain (Support for R&D Projects; BGP18/ 00178).

## Disclosures

None.

## Ethical Approval

The National Research Ethics Service West London committee provided ethical approval (CHD: 07/H0707/105 and 21/WA/0075; dHCP: 14/LO/1169). In accordance with the Declaration of Helsinki, informed written parental consent was obtained before preoperative MRI and confirmed before postoperative MRI.

## Supporting information

Tables S1–S10
