# Supplementary material for: Individual Assessment of Perioperative Brain Growth Trajectories in Infants With Congenital Heart Disease: Correlation With Clinical and Surgical Risk Factors
Source: J Am Heart Assoc. 2023 Jul 8;12(14):e028565. doi: 10.1161/JAHA.122.028565 (PMC10382106; doi:10.1161/JAHA.122.028565)
Supplement: Supplementary file 1 — Tables S1–S10 [file JAH3-12-e028565-s001.pdf]

Supplemental Material

Table S1. Pre to postoperative Z-score slopes by CHD category.

Region	Abnormal streaming of blood change	Left-sided heart lesions change	Right-sided heart lesions change	pFDR
Extracerebral CSF	-0.486 (-0.823- -0.140)	-0.783 (-1.721- -0.398)	-0.406 (-0.478- -0.180)	0.664
Cortical grey matter	-0.303 (-0.524- 0.030)	-0.311 (-0.417- -0.185)	-0.362 (-0.610- -0.201)	0.933
White matter	-0.138 (-0.568- 0.052)	-0.004 (-0.254- 0.089)	-0.264 (-0.284- -0.135)	0.933
Ventricles	0.107 (-0.054- 0.472)	0.057 (-0.345- 0.409)	-0.423 (-0.518- -0.269)	0.664
Cerebellum	-0.457 (-1.496- -0.055)	-0.718 (-1.075- -0.277)	-0.742 (-1.042- -0.666)	0.933
Brainstem	-0.911 (-1.499- -0.127)	-0.687 (-0.933- -0.437)	-0.278 (-0.862- -0.049)	0.664
Left Thalamus	0.012 (-0.563- 0.194)	-0.111 (-0.554- 0.115)	-0.103 (-0.115- -0.020)	0.933
Right Thalamus	-0.275 (-0.966- -0.039)	-0.331 (-0.542- 0.397)	-0.-041 (-0.290- 0.129)	0.933
Left Caudate	0.028 (-0.375- 0.295)	-0.032 (-0.221- 0.301)	0.056 (-0.095- 0.149)	0.664
Right Caudate	-0.155 (-0.721- 0.051)	-0.034 (-0.284- 0.270)	0.283 (0.124- 0.352)	0.933
Left Lentiform	0.480 (0.247- 0.770)	0.039 (-0.141- 0.439)	-0.303 (-0.520- 0.002)	0.315
Right Lentiform	0.050 (-0.472- 0.267)	-0.151 (-0.222- -0.029)	-0.604 (-0.787- -0.400)	0.664
Total Tissue Volume	-0.146 (-0.611- 0.005)	-0.220 (-0.409- -0.064)	-0.392 (-0.527- -0.213)	0.933

CSF = Cerebrospinal Fluid

Table S2. Deviations in Z-scores before and after surgery and in Z-score slope.

Region	Extreme deviations before surgery only, N (%)	Extreme deviations after surgery only, N (%)	Extreme deviations both before and after surgery, N (%)	Significant changes in Z-score slope. N (%)
Extracerebral CSF	>2.6: 3 (8.3)	>2.6: 2 (5.6)	>2.6: 1 (2.8)	>1.65: 1 (2.8)<br><-1.65: 5 (13.9)
Cortical grey matter	0 (0)	<-2.6: 3 (8.3)	0 (0)	<-1.65: 1 (2.8)
White matter	0 (0)	0 (0)	<-2.6: 1 (2.8)	0 (0)
Ventricles	>2.6: 1 (2.8)	>2.6: 3 (8.3)	>2.6: 2 (5.6)	>1.65: 1 (2.8)
Cerebellum	0 (0)	<-2.6: 5 (13.9)	0 (0)	<-1.65: 5 (13.9)
Brainstem	0 (0)	<-2.6: 6 (16.7)	<-2.6: 1 (2.8)	<-1.65: 4 (11.1)
Left Thalamus	0 (0)	<-2.6: 4 (11.1)	<-2.6: 3 (8.3)	<-1.65: 2 (5.6)
Right Thalamus	<-2.6: 1 (2.8)	<-2.6: 2 (5.6)	<-2.6: 2 (5.6)	<-1.65: 1 (2.8)
Left Caudate	0 (0)	<-2.6: 4 (11.1)	<-2.6: 3 (8.3)	0 (0)
Right Caudate	0 (0)	0 (0)	0 (0)	<-1.65: 1 (2.8)
Left Lentiform	<-2.6: 1 (2.8)	0 (0)	0 (0)	0 (0)
Right Lentiform	0 (0)	0 (0)	0 (0)	>1.65: 2 (5.6)

Total Tissue Volume	0 (0)	<-2.6: 2 (5.6)	<-2.6: 1 (2.8)	0 (0)

CSF = Cerebrospinal Fluid

Table S3. Infants with significant deviations in brain development.

Sex	Gestational age at birth (weeks)	Post menstrual age at pre-op scan (weeks)	Post menstrual age at post op scan (weeks)	CHD Diagnosis	Brain injury findings before surgery	Brain Injury findings after surgery	Extreme Deviations before surgery	Extreme Deviations after surgery	Significant change in slope
Male	38.86	39.53	42.71	Coarctation of the aorta	Mild ventricular dilatation	Microhaemorrahges, cerebellar haemorrhage	>2.6: Extracerebral CSF	-	<-1.65: Extracerebral CSF
Female	39.00	39.71	45.14	Coarctation of the aorta	White matter injury: 5 lesions	White matter injury: 1 lesion	>2.6: Extracerebral CSF	-	<-1.65: Extracerebral CSF
Male	38.29	38.57	41.14	TGA	Normal	White matter injury: 4 lesions	>2.6: Extracerebral CSF	<-2.6: Caudate L	<-1.65: Brainstem
Male	38.57	38.71	41.86	Truncus arteriosus (22q11.2 deletion)	White matter injury: 1 lesion Intraventricular haemorrhage	White matter injury: 2 lesions	<-2.6: Caudate L, Lentiform L	<-2.6: Caudate L, Thalamus L, Cerebellum, Brainstem	<-1.65: Thalamus L, Cerebellum, Brainstem
Female	37.57	38.29	42.14	TGA	Normal	Normal	-	>2.6: CSF, Ventricles	>1.65: Extracerebral CSF*, Ventricles*, Lentiform R
Male	38.43	38.71	41.00	TGA	Normal	White matter injury: 4 lesions	-	<-2.6: Cerebellum	<-1.65: Extracerebral CSF^
Male	39.14	39.29	41.71	TGA	Normal	Arterial ischaemic stroke right occipital and left frontal cortex	-	<-2.6: Brainstem, Caudate L	<-1.65: Brainstem, Cerebellum

Female	39.71	40.57	44.71	Coarctation of the aorta	Normal	Normal	-	-	<-1.65: Cortical grey matter, cerebellum, caudate R
Female	37.43		40.43	TGA	Normal	White matter injury: 6 lesions<br>Cerebral sinus venous thrombosis	-	-	<-1.65: Extracerebral CSF
Male	39.57	41.14	42.57	TGA	White matter injury: 1 lesion	Normal	-	-	>1.65: Lentiform R
Female	36.86	37.14	38.86	Coarctation of the aorta	Normal	White matter injury: more than 10 lesions	-	<-2.6: Brainstem, Thalamus L, Thalamus R	<-1.65: Extracerebral CSF, Thalamus R
Male	36.71	40.00	41.71	Coarctation of the aorta	Normal	Normal	-	<-2.6: Cortical grey matter, Cerebellum Caudate L, Thalamus L, Total tissue volume	<-1.65: Cerebellum
Female	38.00	38.14	43.71	Truncus arteriosus	Normal	Normal	-	<-2.6: Cortical grey matter, Cerebellum, Brainstem, Thalamus L, Thalamus R, total tissue volume	<-1.65: Cerebellum, Brainstem

Female	39.00	41.00	42.71	TGA	Infarct in right putamen and left postcentral gyrus	Infarct in right putamen and left postcentral gyrus	>2.6: Ventricles	-	-
Male	37.57	37.57	38.86	Coarctation of the aorta	Mild Cerebellar Haemorrhage	Infarct in Right Striatum	<-2.6: White matter, Brainstem, Thalamus L, Thalamus R, Total Tissue Volume	<-2.6: White matter, Brainstem, Thalamus L, Thalamus R, Total Tissue Volume, Cortical grey matter, Cerebellum, Caudate L	-
Female	39.29	40.86	42.86	Pulmonary atresia	Normal	Normal	<-2.6: Caudate L, Thalamus L, Thalamus R	<-2.6: Caudate L, Thalamus L, Cerebellum, Brainstem	-
Male	40.0	40.71	41.86	Pulmonary atresia	Mild Ventricular Dilatation	Mild Ventricular dilatation	>2.6: Extracerebral CSF, Ventricles<br><br><-2.6: Caudate L	>2.6: Extracerebral CSF, Ventricles<br><br><-2.6: Caudate L	-

Female	38.43	39.00	40.57	TGA	Mild Ventricular Dilatation	Mild Ventricular Dilatation	>2.6: Ventricles<br><br><-2.6: Thalamus L, Thalamus R	>2.6: Ventricles<br><br><-2.6: Thalamus L, Thalamus R	-
Female	38.57	39.57	40.71	Coarctation of the aorta	White matter injury: 8 lesions	White matter injury: 8 lesions<br>Infarct right thalamus	-	>2.6: Extracerebral CSF	-
Male	38.29	39.43	44.29	TGA	Normal	Normal	-	>2.6: Ventricles	-
Male	39.00	39.57	45.57	TGA	Normal	White matter injury: 1 lesions	-	>2.6: Ventricles	-

CSF = Cerebrospinal Fluid

Table S4. Extreme deviations before and after surgery by CHD group.

	Before and after Surgery	Before surgery only	After surgery only	Significant changes
Abnormal Streaming of flow (N=20)	3 (15)	0 (0)	2 (10)	8(40)*
Left heart abnormalities (N=12)	1 (8.3)	2 (16.7)	2 (16.7)	5 (41)
Right heart abnormalities (N=4)	2 (50)	0 (0)	0 (0)	0 (0)
p_{FDR}	1.00	0.762	1.00	0.817

*Significant at threshold 2.3 (n=2) and 2.6 (n=1)

Table S5. Relationship between timing of MRI and surgery and Z-score slope.

	**Postnatal age at surgery**		**Timing of postoperative MRI**		**Interval between pre and postoperative MRI**	
**Region**	**ρ**	**p_{FDR}**	**ρ**	**p_{FDR}**	**ρ**	**p_{FDR}**
Extracerebral CSF	-0.174	0.599	-0.106	0.747	-0.212	0.720
Cortical grey matter	-0.381	0.237	0.038	0.899	0.035	0.844
White matter	-0.399	0.126	-0.175	0.599	-0.183	0.720
Ventricles	-0.069	0.861	0.133	0.747	0.103	0.803
Cerebellum	-0.333	0.173	-0.009	0.959	-0.125	0.803
Brainstem	**-0.481**	**0.042**	0.115	0.747	-0.059	0.803
Right Caudate	-0.232	0.864	0.071	0.804	-0.008	0.720
Left Caudate	-0.029	0.903	0.177	0.599	0.229	0.803
Right Lentiform	**-0.477**	**0.042**	0.157	0.622	-0.073	0.720
Left Lentiform	-0.268	0.331	0.325	0.173	0.235	0.803
Right Thalamus	-0.348	0.162	0.048	0.884	-0.178	0.720
Left Thalamus	-0.219	0.467	0.104	0.747	-0.066	0.803
Total Tissue Volume	-0.403	0.126	-0.055	0.884	-0.060	0.803

Results in bold are significant.

CSF = Cerebrospinal Fluid

Table S6. Relationship between change in weight and head circumference Z-score and Z-score slope.

	Weight Z-score		Head Circumference Z-score	
**Z-score slope**	**ρ**	**p_{FDR}**	**ρ**	**p_{FDR}**
Extracerebral CSF	0.274	0.323	0.207	0.422
Cortical grey matter	0.283	0.323	0.132	0.593
White Matter	0.061	0.734	-0.155	0.589
Ventricles	0.378	0.130	0.175	0.523
Cerebellum	0.247	0.348	0.072	0.734
Brainstem	0.376	0.130	0.202	0.422
Right Caudate	0.439	0.088	0.097	0.593
Left Caudate	0.442	0.088	0.124	0.686
Right Lentiform	0.206	0.422	0.137	0.591
Left Lentiform	-0.062	0.734	-0.264	0.323
Right Thalamus	0.275	0.323	0.144	0.589
Left Thalamus	0.423	0.088	0.234	0.382
Total Tissue Volume	0.275	0.130	0.069	0.734

CSF = Cerebrospinal Fluid

Table S7. Relationship between Z-score slope and preoperative creatinine covarying for age at sample.

**Z-score slope**	**ρ**	**p_{FDR}**
Extracerebral CSF	-0.094	0.712
Cortical grey matter	-0.308	0.175
White matter	-0.230	0.286
Ventricles	-0.066	0.774
Cerebellum	-0.345	0.128
Brainstem	**-0.503**	**0.033**
Right Caudate	**-0.434**	**0.039**
Left Caudate	**-0.476**	**0.033**
Right Lentiform	-0.032	0.859
Left Lentiform	0.173	0.435
Right Thalamus	**-0.436**	**0.038**
Left Thalamus	-0.279	0.189
Total Tissue Volume	-0.279	0.189

Results in bold are significant

CSF = Cerebrospinal Fluid

Table S8. Preoperative, postoperative and new postoperative injuries across the cohort.

	Preoperative MRI injuries	Postoperative MRI injuries	New injuries on postoperative MRI
Infants with preoperative injury and no new injury on postoperative MRI			
1	WMI	none	none
2	WMI	none	none
3	WMI	WMI	none
4	WMI	WMI	none
5	WMI	WMI	none
6	WMI + small thalamic infarct	WMI + small thalamic infarct	none
7	Infarct in right putamen and left postcentral gyrus	Infarct in right putamen and left postcentral gyrus	none
8	Mild ventricular dilatation	Mild ventricular dilatation	none
9	Mild ventricular dilatation	Mild ventricular dilatation	none
Infants with no preoperative injury and new injury on postoperative MRI			
10	none	Unilateral small cerebellar hemorrhages*	Unilateral small cerebellar hemorrhages*
11	none	WMI	WMI
12	none	WMI	WMI

13	none	WMI	WMI
14	none	WMI	WMI
15	none	WMI	WMI
16	none	WMI	WMI
17	none	Arterial ischemic stroke right occipital and left frontal cortex	Arterial ischemic stroke right occipital and left frontal cortex
18	none	WMI + CSVT	WMI + CSVT
Infants with preoperative injury and new injury on postoperative MRI			
19	WMI	WMI	WMI
20	WMI	WMI	WMI
21	WMI + intraventricular hemorrhage	WMI	WMI
22	WMI + small unilateral cerebellar hemorrhage + small germinal layer hemorrhage + small venous infarct	WMI + small unilateral cerebellar hemorrhage + small germinal layer hemorrhage + small venous infarct	WMI
23	WMI	WMI + infarct in right thalamus	WMI + infarct in right thalamus
24	Left MCA infarct + WMI	Left MCA infarct + WMI + CSVT	WMI + CSVT
25	Small unilateral cerebellar hemorrhage	Small unilateral cerebellar hemorrhage + WMI + infarct in right striatum	WMI + infarct in right striatum
26	Small unilateral cerebellar microhemorrhages + posterior fossa hemorrhage	Small putamen infarct	Small putamen infarct

No injury on pre or postoperative MRI			
27 - 36	none	none	none

*Cerebellar hemorrhages were identified on an additional preoperative MRI

CSVT = cerebral sinus venous thrombosis; WMI = white matter injury; MCA = middle cerebral artery

Table S9. Relationship between the presence of pre-operative, post-operative and any new postoperative brain injury and white matter injury (WMI) with change in Z-score slope.

	Preoperative injury (pFDR)	Postoperative injury (pFDR)	New postoperative injury (pFDR)	Preoperative WMI (pFDR)	Postoperative WMI (pFDR)	New postoperative WMI (pFDR)
Extracerebral CSF	0.95	0.95	0.56	0.97	>0.99	0.74
Cortical grey matter	0.95	0.94	0.81	0.97	>0.99	0.92
White Matter	0.95	0.76	0.81	0.97	>0.99	0.92
Ventricles	0.95	0.94	0.69	0.97	>0.99	0.92
Cerebellum	0.95	0.92	0.56	0.97	>0.99	0.92
Brainstem	0.95	0.56	0.49	0.97	>0.99	0.74
Right Caudate	0.95	0.94	0.23	0.97	>0.99	0.74
Left Caudate	0.95	0.94	0.80	0.97	>0.99	0.92
Right Lentiform	0.95	0.94	0.56	0.97	>0.99	0.92
Left Lentiform	0.95	0.94	0.81	0.97	>0.99	0.74
Right Thalamus	0.95	0.76	0.49	0.97	>0.99	0.74
Left Thalamus	0.95	0.15	0.56	0.97	>0.99	0.92
Total Tissue Volume	0.95	0.58	0.81	0.97	>0.99	0.92

CSF = Cerebrospinal Fluid

Table S10. Relationship between Z-score slope pre to postoperatively and time on bypass, and circulatory arrest, correcting for time on PICU.

	Cardiopulmonary bypass time		Circulatory Arrest time	
**Z-score slope**	**ρ**	**p_{FDR}**	**ρ**	**p_{FDR}**
Extracerebral CSF	-0.162	0.478	-0.076	0.703
Cortical grey matter	-0.080	0.703	-0.232	0.361
White matter	-0.162	0.478	0.460	0.061
Ventricles	-0.185	0.478	-0.124	0.580
Cerebellum	-0.164	0.478	0.310	0.267
Brainstem	**-0.518**	**0.026**	0.242	0.350
Right Caudate	**-0.587**	**0.009**	0.179	0.478
Left Caudate	-0.247	0.350	0.297	0.268
Right Lentiform	-0.011	0.949	0.314	0.257
Left Lentiform	0.091	0.695	0.137	0.555
Right Thalamus	-0.390	0.335	0.426	0.087
Left Thalamus	-0.261	0.109	0.285	0.279
Total Tissue Volume	-0.211	0.412	0.389	0.109

PICU = Paediatric Intensive Care Unit; CSF = Cerebrospinal Fluid
